# Supplementary material for: Development of novel g-SSR markers in guava (Psidium guajava L.) cv. Allahabad Safeda and their application in genetic diversity, population structure and cross species transferability studies
Source: PLoS One. 2020 Aug 17;15(8):e0237538. doi: 10.1371/journal.pone.0237538 (PMC7431106; doi:10.1371/journal.pone.0237538)
Supplement: Attachment — Submitted filename: Reply to Reviewers comments.docx [file pone.0237538.s001.docx]

**Response to Reviewers**

**Reviewer #1:** Manuscript has been written quite satisfactorily in light of the above literature. However, there are few typos in the manuscript, especially, regarding space before/ after comma. Further, 'Introduction', especially, first two paragraphs may be drastically shortened.

**Reply:** Authors are highly thankful to reviewer to his encouraging comments and critical review of the manuscript. All the typos especially the space before and after comma has been corrected. The Introduction part has been modified and reduced as suggested by the reviewer.

**Reviewer #2:** I would like to appreciate the authors (researchers) for their sincere efforts in adding useful genomic information to guava crop. They have used sound methodologies, software and statistical analysis for concluding their results.
Manuscript is well written. Most of the information provided by them is self-explanatory.

**Reply:** Authors would like to thank reviewer for his kind words and encouragement.

One correction in abstract, line no.31 It is AMOVA, mistakenly written as AMOA

**Reply:** The correction suggested has been incorporated in the manuscript.
